# Cholesterol biosynthesis modulates differentiation in murine cranial neural crest cells

**DOI:** 10.1038/s41598-023-32922-9

**Published:** 2023-05-01

**Authors:** Florencia Pascual, Mert Icyuz, Peer Karmaus, Ashley Brooks, Elizabeth Van Gorder, Michael B. Fessler, Natalie D. Shaw

**Affiliations:** 1 Clinical Research Branch, National Institute of Environmental Health Sciences, 111 TW Alexander Drive, MD D3-02, Research Triangle Park, NC 27709 USA; 2 Immunity, Inflammation, and Disease Laboratory, National Institute of Environmental Health Sciences, 111 TW Alexander Drive, Research Triangle Park, NC USA; 3 Biostatistics and Computational Biology Branch, National Institute of Environmental Health Sciences, 111 TW Alexander Drive, Research Triangle Park, NC USA

**Keywords:** Differentiation, Cell biology

## Abstract

Cranial neural crest cells (cNCC) are a multipotent embryonic cell population that give rise to a diverse set of cell types. These cells are particularly vulnerable to external metabolic stressors, as exemplified by the association between maternal hyperglycemia and congenital malformations. We were interested in studying the effect of various concentrations of glucose and pyruvate on cNCC metabolism, migration, and differentiation using an established murine neural crest cell model (O9-1). We unexpectedly observed a pattern of gene expression suggestive of cholesterol biosynthesis induction under glucose depletion conditions in O9-1 cells. We further showed that treatment with two different cholesterol synthesis inhibitors interfered with cell migration and differentiation, inhibiting chondrogenesis while enhancing smooth muscle cell differentiation. As congenital arhinia (absent external nose), a malformation caused by mutations in *SMCHD1*, appears to represent, in part, a defect in cNCC, we were also interested in investigating the effects of glucose and cholesterol availability on *Smchd1* expression in O9-1 cells. *Smchd1* expression was induced under high glucose conditions whereas cholesterol synthesis inhibitors decreased *Smchd1* expression during chondrogenesis. These data highlight a novel role for cholesterol biosynthesis in cNCC physiology and demonstrate that human phenotypic variability in *SMCHD1* mutation carriers may be related, in part, to *SMCHD1*’s sensitivity to glucose or cholesterol dosage during development.

## Introduction

Neural crest cells (NCCs) are a transient embryonic cell population derived from the ectoderm that give rise to a diverse set of cell types. During embryonic development, migrating NCCs traverse diverse environments with unique nutrients and localized activation of enzymes that can impact their genetic programming and physiology^1^. Studies examining the effect of substrate availability perturbations show that the spatiotemporal regulation of development is in part driven by changes in metabolism^2^. Metabolic changes in NCC are temporally associated with, and may in fact stimulate, critical steps in NCC ontogeny such as proliferation, migration, and differentiation^3^. In addition, NCC appear to be particularly vulnerable to external metabolic stressors, hyperglycemia being a prime example. Gestational diabetes is associated with a higher risk of congenital malformations affecting tissues and organs derived from NCC (e.g., cardiovascular, skeletal, and central nervous systems), suggesting that maternal hyperglycemia is highly toxic to NCC^4–7^. Indeed, early in vitro studies demonstrated that high glucose culture conditions inhibit rat cNCC proliferation and migration due to reactive oxygen species overproduction^8^. More recent work in the chick has further shown that exposure to high glucose upregulates apoptosis and ERK-mediated autophagy in developing cNCC^9^ and suppresses embryonic stem cell differentiation into a neuronal lineage^10^. There have been no studies, however, to determine how nutrient availability affects NCC physiology using the O9-1 cell line, a multipotent line derived from mouse embryonic NCCs^11^.

Defects in the ontogeny, migration, and/or differentiation of NCC give rise to a set of conditions called neurocristopathies. Bosma arhinia microphthalmia syndrome (BAMS) is an extremely rare, severe congenital malformation that appears to reflect a primary defect of the cranial NCC^12^, cranial placode cells^13^ or their interaction. BAMS consists of the clinical triad of arhinia (absent nose), eye defects, and hypogonadism^14^ and is caused by mutations in the gene Structural Maintenance of Chromosomes Flexible Hinge Domain-containing 1 (*SMCHD1*)^15,16^. However, the presence of incomplete penetrance and variable expressivity in multiplex families suggests that other in utero factors may influence SMCHD1 expression or function. We hypothesized that nutrient availability could be one such factor. Gestational diabetes has not been reported in BAMS pregnancies, however, BAMS is likely to be underreported (< 100 cases reported in the past century^15^), guidelines for diagnosing gestational diabetes vary from country to country and have become stricter over time, and it has recently been recognized that maternal hyperglycemia is linearly associated with perinatal risk without an obvious threshold^17^. Thus, using the O9-1 model system, we also investigated the effect of nutrient availability on *Smchd1* expression.

## Results

### Glucose availability affects O9-1 cNCC physiology

We hypothesized that different metabolic conditions would impact cNCC physiology. We studied the effect of 4 culture conditions: high glucose (HG = 25 mM glucose, 1 mM pyruvate), control glucose (CG = 5.55 mM glucose, 1 mM pyruvate), no glucose (NG = 0 mM glucose, 1 mM pyruvate), and no glucose with 2 × pyruvate (NG2P = 0 mM glucose, 2 mM pyruvate). While cell culture protocols frequently utilize HG conditions to maximize proliferation, the CG condition was selected to best represent the physiology of the developing embryo^18^. The NG condition was chosen to determine if cNCC could use alternative metabolic substrates such as pyruvate; pyruvate was of particular interest given its position at the crossroads of multiple pathways in carbon metabolism and demonstrated roles in embryonic genome activation^19^ and NCC physiology^3,20,21^.

**Figure 1:**
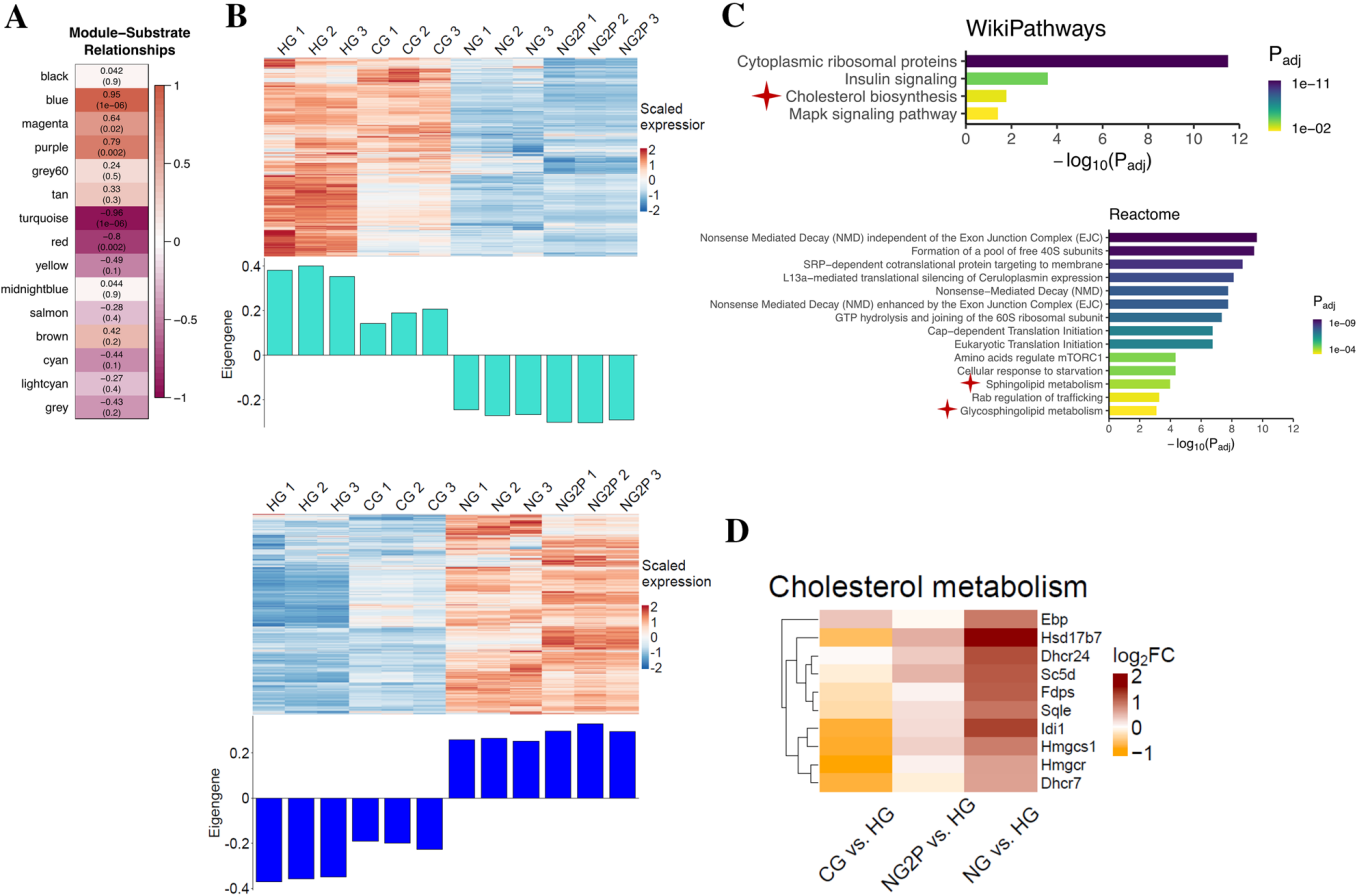
Glucose availability impacts the cNCC transcriptome. (**A**) Pearson correlation values between module gene expression level and substrate availability; high glucose (HG = 25 mM glucose, 1 mM pyruvate), control glucose (CG = 5.55 mM glucose, 1 mM pyruvate), no glucose (NG = 0 mM glucose, 1 mM pyruvate), and no glucose with 2 × pyruvate (NG2P = 0 mM glucose, 2 mM pyruvate) with adjusted p-values (in parentheses) are shown in each bin; correlation of 1 or -1 indicates a strong positive or negative relationship, respectively. (**B**) Heatmaps and bar plots display scaled gene expression and eigengene values for the turquoise and blue modules. (**C**) Pathway analysis of blue module genes filtered for module membership > 0.6. (**D**) Heatmap of genes involved in cholesterol biosynthesis.

**Figure 2:**
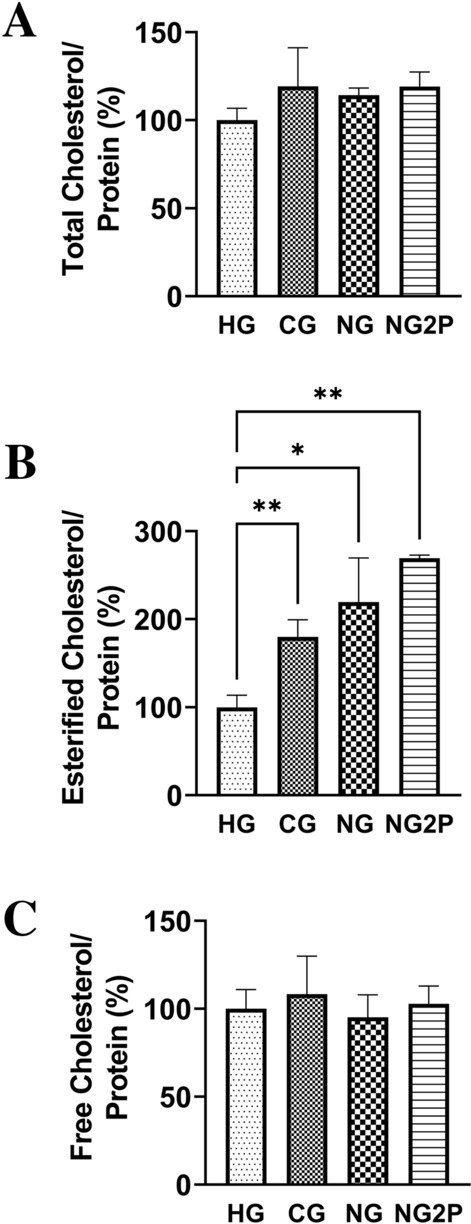
Glucose availability regulates esterified cholesterol levels in cNCC. Total, esterified, and free cholesterol levels normalized to protein in cNCC cultured in HG, CG, NG, and NG2P conditions. n = 3 per group. Values are shown as the mean ± SD. *p < 0.05, **p < 0.01.

To determine how different glucose conditions (HG, CG, NG, NG2P) affect gene expression in mouse cNCC, a weighted gene correlation network analysis (WGCNA) was employed to interpret RNA-Seq data. WGCNA constructs gene co-expression networks by taking into account correlation patterns among genes across samples^22^. Hierarchical clustering is then used to identify modules, or networks of genes with highly correlated gene expression. These modules can then be related to other traits (here, glucose concentration) and interrogated for functional enrichment. Modules of interest are selected based on either mean gene significance, module membership (gene connectivity within a module), relationship to a trait, or biological pathways. To select modules of interest, we first considered the strength of module membership (connectivity > 0.6). Given that our experimental design included a gradient of glucose concentrations, we then chose to explore the two modules where there was also a linear gradient in expression change from HG to CG to NG to NG2P. Sixteen modules of co-expressed genes under different glucose conditions were identified. In the turquoise module, gene expression decreased across conditions, whereas in the blue module, gene expression increased across conditions (Fig. 1A, B, Supplementary Table S1). Overenrichment analysis of the turquoise model revealed pathways associated with cell cycle and DNA repair (Supplementary Fig. S1), whereas analysis of the blue module unexpectedly revealed cholesterol biosynthesis, sphingolipid, and glycosphingolipid metabolism (Fig. 1C, Supplementary Fig. S1). Glucose and glucose-derived metabolites provide raw materials for cholesterol synthesis and regulate cholesterol biosynthetic enzymes and uptake^23^. Glucose depletion would therefore be expected to downregulate cholesterol biosynthesis; however, members of the cholesterol biosynthesis pathway including *Hmgcr*, the rate-limiting enzyme in cholesterol synthesis, and *Hmgcs1*, which catalyzes the production of 3-hydroxy-3-methylglutaryl-CoA (HMG-CoA)^24,25^, showed increased expression under NG and NG2P conditions compared to HG (Fig. 1D). Emopamil binding protein (Ebp), which plays a key role in the final stage of cholesterol biosynthesis, was also upregulated in CG vs. HG conditions (Fig. 1D). We next directly measured free cholesterol, esterified cholesterol, and total (i.e., the sum of free plus esterified) cholesterol levels under the various glucose culture conditions. No significant differences were seen in free or total cholesterol, whereas there was lower esterified cholesterol in HG compared with CG, NG, and NG2P conditions (Fig. 2A–C).

### Pharmacological inhibition of cholesterol biosynthesis in cNCCs enhances programmed cell death

**Figure 3:**
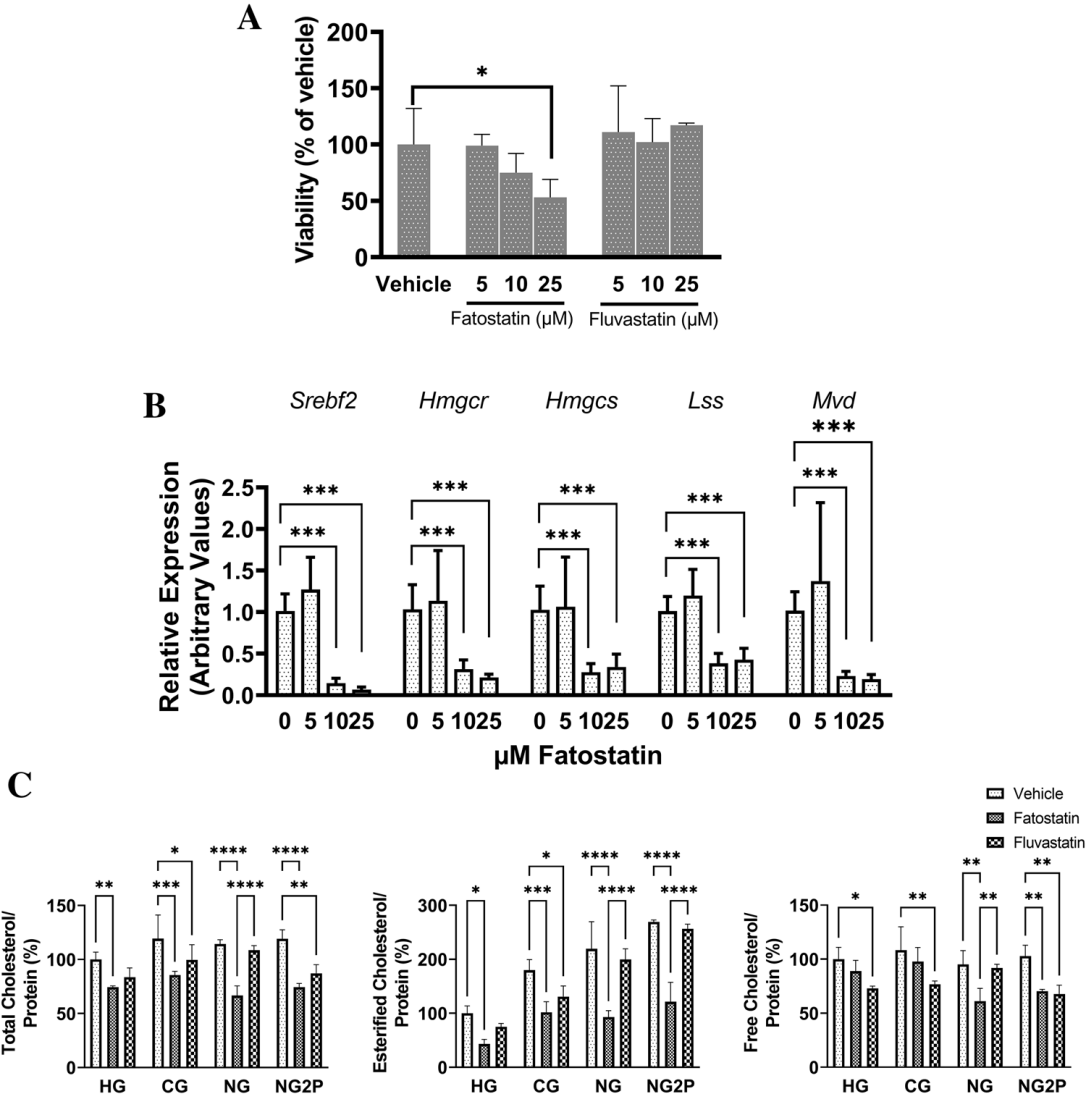
Cholesterol synthesis inhibition in O9-1 cNCC. (**A**) Viability of O9-1 cNCC cultured for 48 h in the presence of fatostatin or fluvastatin at 5, 10, and 25 µM compared to vehicle (“0”) conditions. n = 3 per group. (**B**) Gene expression levels of *Srebf2*, *Hmgcr*, *Hmgcs*, *Lss*, *Mvd* normalized to *18S* levels in cells cultured in HG treated with 0 (vehicle), 5, 10, and 25 µM fatostatin. (**C**) Total, esterified, and free cholesterol levels normalized to protein in cNCC cultured in HG, CG, NG, and NG2P conditions in the absence (vehicle) or presence of 10 µM fatostatin of fluvastatin. n = 3 per group. Values are shown as the mean ± SD. *p < 0.05, **p < 0.01, ***p < 0.001, ****p < 0.0001.

We set out to examine the significance of cholesterol biosynthesis in cNCC physiology by using two drugs that are structurally dissimilar and that impact the cholesterol biosynthetic pathway at different points. Fatostatin is a small molecule cholesterol synthesis inhibitor that blocks the activation of SREBP-1 and -2, master regulators of cholesterol and fatty acid synthesis^26^. Fluvastatin directly inhibits HMG-CoA reductase, the rate-limiting enzyme in cholesterol biosynthesis^27^. Both drugs have also been used to inhibit cholesterol synthesis in neuroblastoma, a NCC-derived malignancy. To identify the ideal working concentration of fatostatin in O9-1 cells, we first measured changes in cholesterol synthesis gene expression and cell viability. We chose a range of concentrations (5 to 25 µM) based on previous experiments conducted in embryonic cells^28^. We identified concentrations of fatostatin and fluvastatin that were nontoxic to cNCC via the Incucyte live-cell viability assay based on cell confluency (Fig. 3A). Fatostatin significantly reduced O9-1 cNCC viability at 25 µM. Fatostatin dramatically reduced *Srebf2*, *Hmgcr*, *Hmgcs1*, *Lss*, and *Mvd* mRNA levels at 10 µM and 25 µM after 48 h of treatment (Fig. 3B). Based on these results, we selected 10 μM as the minimal concentration that would elicit cholesterol synthesis inhibition without impairing cell viability for further studies. To validate the observed suppression of cholesterol biosynthesis by fatostatin and fluvastatin, we directly measured cholesterol levels using the Amplex Red assay in O9-1 cells in HG, CG, NG, and NG2P conditions. In the presence 10 µM fatostatin, total and esterified cholesterol levels were significantly lowered in all glucose conditions and free cholesterol levels were decreased in NG and NG2P conditions (Fig. 3C). In the presence of 10 µM fluvastatin, total cholesterol levels were lower in CG and NG2P, esterified cholesterol levels were lower in only CG. Free cholesterol levels were decreased in HG, CG, and NG2P after fluvastatin treatment. Thus, fatostatin was more potent in suppressing total and esterified cholesterol levels than fluvastatin presumably because, by blocking SREBP-2, it has the potential to impact the entire cholesterol biosynthetic pathway.

**Figure 4:**
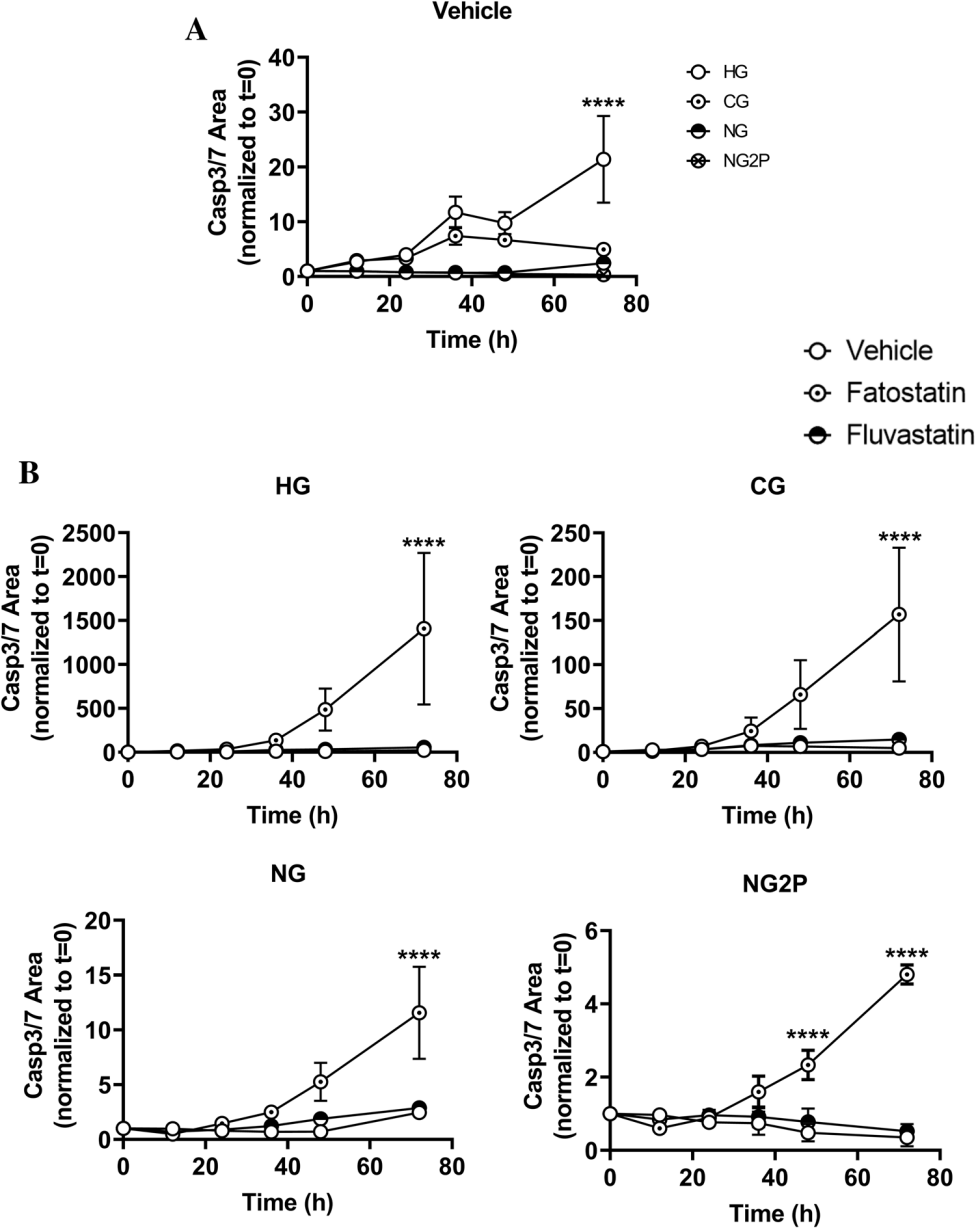
Glucose-mediated regulation of cholesterol metabolism may play a role in cNCC programmed cell death. (**A**) Apoptosis in cNCC cultured in HG, CG, NG, and NG2P conditions plotted as a function of glucose availability to examine the effects of cholesterol synthesis inhibition, was measured via the Incucyte live-cell Casp3/7 apoptosis assay. n = 6 wells/condition; multiple images per well were collected for 3d. (**B**), Apoptosis in cNCC cultured in HG, CG, NG, and NG2P conditions in the absence (vehicle) or presence of 10 μM fatostatin or fluvastatin was measured via the Incucyte live-cell Casp3/7 apoptosis assay. Each bar represents means ± SD. ****p < 0.0001.

Programmed cell death in cNCC is central to craniofacial patterning/shaping^29^. Glucose availability–specifically, high glucose-mediated increases in reactive oxygen species–has been shown to influence cNCC apoptosis^9^. Furthermore, studies have shown both fatostatin and fluvastatin, a direct inhibitor of HMG-CoA reductase, to possess apoptotic effects^30–33^. We were therefore interested in examining the effect of cholesterol inhibitors on cNCC susceptibility to apoptosis under varying glucose conditions. As expected, increased glucose led to a ~ 20-fold increase in apoptosis that reached statistical significance at the latest time point in the absence of cholesterol synthesis inhibitors (Fig. 4A). Examination of apoptosis in the presence of cholesterol synthesis inhibitors revealed that only fatostatin treatment resulted in increased apoptosis (no appreciable difference between fluvastatin and vehicle conditions), and its effect was independent of glucose (Fig. 4B).

### Cholesterol synthesis inhibitors impact cNCC migration

**Figure 5:**
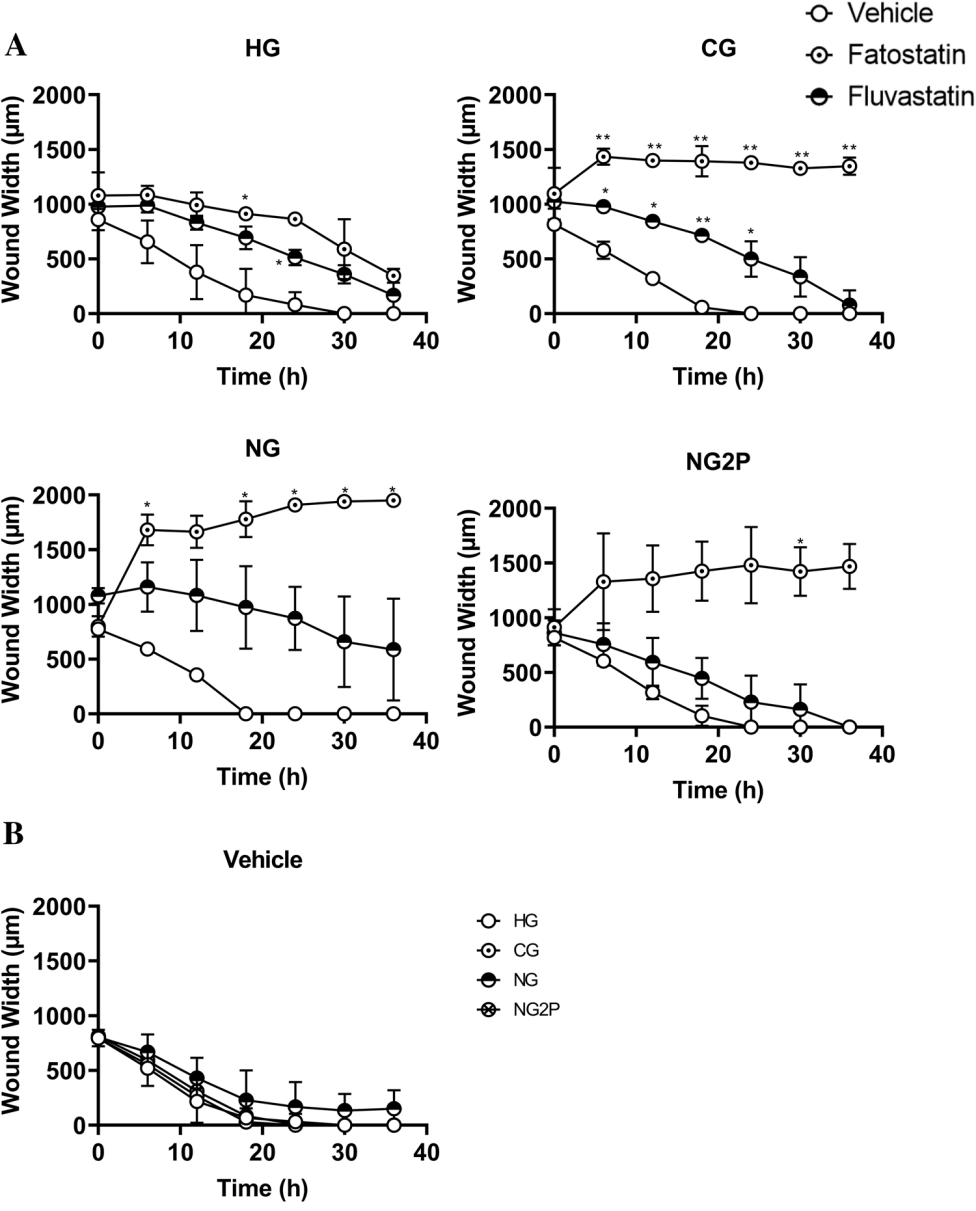
Cholesterol biosynthesis plays a role in cNCC migration. (**A**) Cell migration of cNCC cultured in HG, CG, NG, and NG2P conditions in the absence (vehicle) or presence of 10 μM fatostatin or fluvastatin was examined in an Incucyte live-cell analyzer via a scratch-wound assay. n = 4 per group; multiple images per well were collected every hour for 36 h. Panel (**B**) demonstrates same data in the absence of cholesterol synthesis inhibitors to highlight the effect of glucose availability. Each data point represents means ± SD. *p < 0.05, **p < 0.01, ***p < 0.001, ****p < 0.0001.

As migration from the neural tube is a critical part of cNCC ontogeny, we asked whether blockade of cholesterol synthesis affects cNCC migration (Fig. 5A, B). Migration was measured via a conventional scratch-wound assay in which cells are allowed to reach full confluency before a wound is introduced in the cell monolayer to induce cellular polarization and migration into the resulting space (Supplementary Fig. S2). Wound width was significantly higher in the presence of fatostatin in CG and NG conditions, consistent with decreased migratory ability. Fluvastatin treatment also resulted in increased wound width compared to vehicle treatment in CG and NG; however, this effect achieved statistical significance only in the CG condition. Glucose concentration alone did not affect O9-1 cNCC migration (Fig. 5B).

### O9-1 cNCC differentiation may be regulated by cholesterol

cNCC are multipotent stem cells that give rise to a number of cell types during development, including cranial neurons, glia, smooth muscle cells, osteoblasts, and chondrocytes^30,34,35^. Furthermore, mutations in cholesterol synthesis pathway genes have been associated with facial dysmorphia that results from aberrant WNT signaling-mediated modulation of chondrocyte differentiation^43,44^.

**Figure 6:**
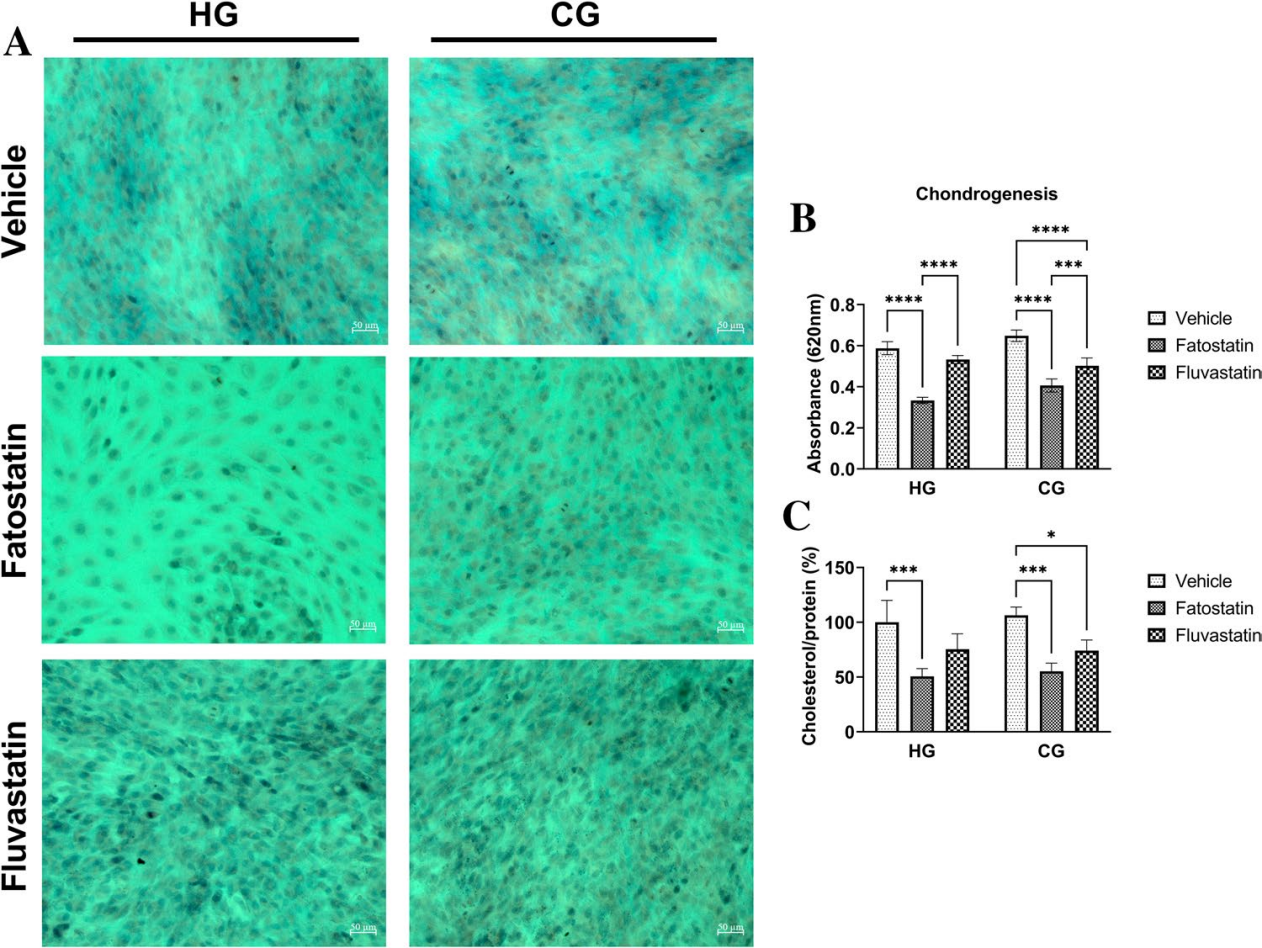
Decreased cholesterol synthesis shifts cNCC terminal fate away from chondrogenesis. (**A**) Alcian blue staining of cNCC cultured in chondrogenesis medium in HG and CG conditions in the presence of vehicle or 5 µM fatostatin and fluvastatin. Scale bars are 50 µM. (**B**) Spectrophotometric quantification of panel A images (4cm^2^ sample wells, n = 5 per group). (**C**) Total cholesterol levels normalized to protein in cNCC cultured in chondrogenesis medium under the specified conditions (n = 3 per group). Each bar represents mean ± SD. *p < 0.05, **p < 0.01, ***p < 0.001, ****p < 0.0001.

To assess whether cholesterol levels affect the potential of cNCC to differentiate into chondrocytes^36,37^, we performed a chondrogenesis assay with O9-1 cells under HG and CG conditions as we were particularly interested in comparing supraphysiologic (akin to hyperglycemia) and physiologic (euglycemic) conditions (Fig. 6). O9-1 cells were cultured in osteogenic differentiation medium for 3 days prior to culture in chondrogenic differentiation medium for 7 days, as previously described^35^. Treatment with 10 µM cholesterol synthesis inhibitors throughout the 10-day differentiation process resulted in disparate cellular confluency across the different glucose conditions; all differentiation experiments were therefore conducted in the presence of 5 µM fatostatin and fluvastatin. Quantification of Alcian blue–used to specifically stain acidic polysaccharides present in cartilage– showed that fatostatin significantly reduced differentiation into chondrocytes in both HG and CG conditions, while fluvastatin reduced differentiation in the CG condition (Fig. 6B). Total cholesterol levels (measured at the end of chondrogenesis) reflected a similar trend: cholesterol was decreased by fatostatin treatment under both HG and CG conditions compared to vehicle, while fluvastatin treatment resulted in decreased cholesterol only under the CG condition, consistent with a role of cholesterol in chondrogenesis (Fig. 6C).

**Figure 7:**
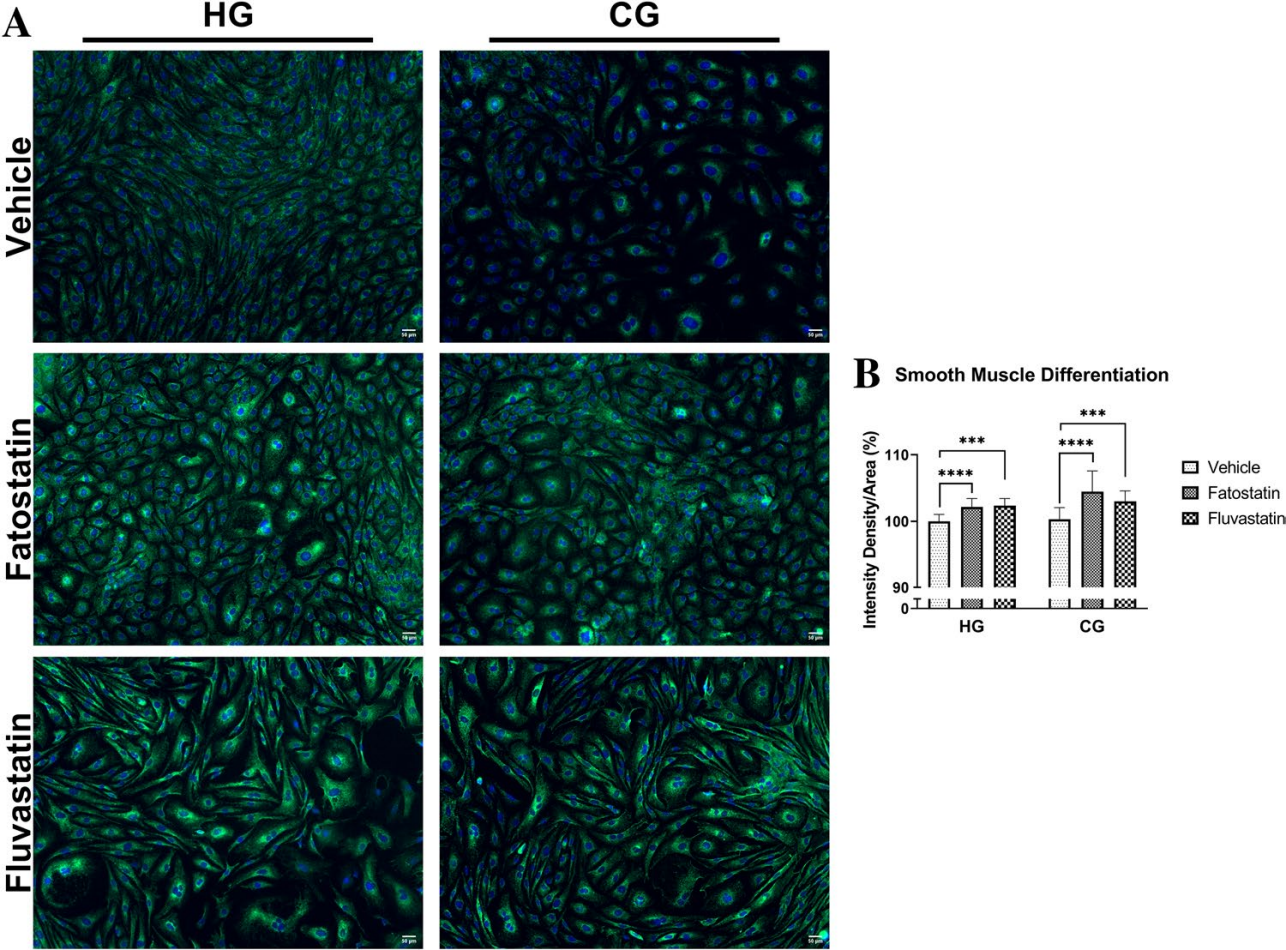
Cholesterol synthesis inhibition favors smooth muscle terminal fates. (**A**) Smooth muscle actin immunostaining of cNCC cultured in HG and CG conditions in the presence of vehicle, fatostatin, and fluvastatin (5 µM). Scale bars are 50 µM. (**B**) Quantification of fluorescence intensity density/area of cNCC stained with smooth muscle actin depicted in panel A. n = 5 frames per group. Each bar represents means ± SD. ***p < 0.001, ****p < 0.0001.

We also examined the capacity of cNCC to differentiate into smooth muscle cells in O9-1 cells cultured under HG and CG conditions in the absence or presence of 5 μM fatostatin and fluvastatin (Fig. 7A). Quantification of smooth muscle actin immunofluorescence showed that blocking cholesterol synthesis via treatment with either fatostatin or fluvastatin significantly increased differentiation into smooth muscle cells in both HG and CG conditions (Fig. 7B).

### Glucose and cholesterol availability and *Smchd1* expression

**Figure 8:**
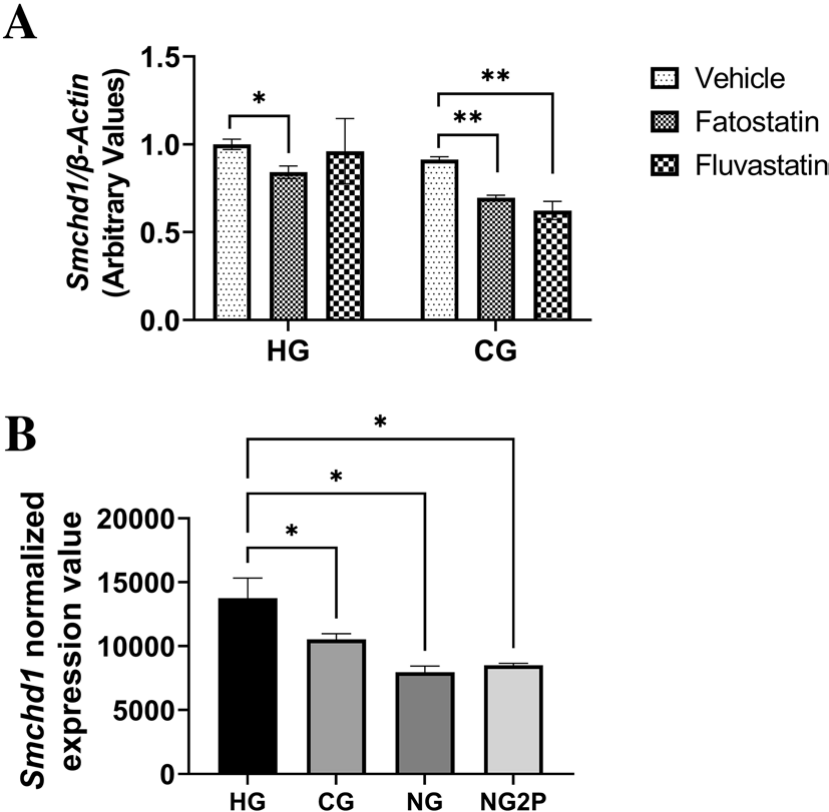
High glucose levels increase *Smchd1* expression in cNCC. (**A**), *Smchd1* gene expression levels normalized to *β-actin* levels in cNCC cultured in chondrogenesis medium under the specified conditions (n = 3 per group). (**B**), *Smchd1* normalized gene expression levels (obtained from RNA-seq study) in cNCC cultured in HG, CG, NG, and NG2P (n = 3 per group). Each bar represents means ± SD. *p < 0.05, **p < 0.01.

Turning our attention back to SMCHD1, we asked whether glucose concentration alone and cholesterol synthesis inhibition during chondrogenesis might affect *Smchd1* expression (Fig. 8A). Treatment with fatostatin decreased *Smchd1* mRNA expression in both CG and HG conditions, while fluvastatin treatment affected *Smchd1* expression only in the CG condition. Glucose concentration during chondrogenesis did not affect *Smchd1* expression (Fig. 8A). At baseline, however, *Smchd1* mRNA expression was significantly increased in HG compared to CG, NG, and NG2P conditions (Fig. 8B) in O9-1 cells.

## Discussion

NCCs are a population of early embryonic, multipotent progenitor cells unique to vertebrates^38^. They arise from the embryonic ectoderm and undergo an epithelial to mesenchymal transition as they delaminate and migrate throughout the body^39^, contributing to a wide variety of structures including, but not limited to, craniofacial cartilage and bone, smooth muscle, melanocytes, myofibroblasts, peripheral/enteric neurons, and glial cells^40^. The extensive migratory capacity and multipotency of NCC is coupled with–and may depend on– a rewiring of metabolism, which not only serves to meet these cells’ unique energy demands but may also provide metabolites that modulate gene transcription and thereby influence differentiation. Building on prior studies pointing to a central role for glycolysis in this process^3,8–10,20,41–44^, we set out to elucidate how perturbations in glucose availability affect cNCC physiology. Our RNA-seq WGCNA and cholesterol data suggest that under elevated glucose conditions–such as during gestational diabetes, for instance– cholesterol esterification is suppressed.

We further investigated the biological relevance of cholesterol synthesis in processes central to cNCC function. We achieved significant downregulation of cholesterol biosynthetic genes and cellular levels of cholesterol via pharmacological inhibition of cholesterol biosynthesis without compromising cell viability. We observed decreased cell migration (increased wound width) with both fatostatin (CG and NG condition) and fluvastatin (CG and trend with NG condition). Interestingly, cholesterol synthesis inhibition did not significantly alter migration of O9-1 cells cultured under glucose depletion conditions when supplemented with 2 × pyruvate. Although high glycolytic flux has been previously shown to be required for proper NCC migration^45^, our results are also suggestive of an interplay between pyruvate and the cholesterol-mediated regulation of cNCC migration that warrants further investigation. Finally, in the presence of fatostatin (CG and HG condition) and fluvastatin (CG condition only), we observed a diminished ability of cNCC to differentiate into chondrocytes and a shift towards the formation of smooth muscle cells. Disruption of cholesterol biosynthesis results in defective Sonic-Hedgehog signaling, which has critical roles in cNCC proliferation and survival^46,47^. Moreover, cholesterol-rich lipid rafts are known to regulate canonical Wnt signaling, which is involved in cell proliferation and cell fate determination during embryonic development^48^. Indeed, Castro et al. showed that cholesterol synthesis inhibition in zebrafish led to facial defects that could be rescued by a Wnt agonist^37^. Wnt signaling is important in both chondrogenesis^49^ and smooth muscle development^50^. Thus, it is possible that a change in Wnt signaling favors differentiation toward a smooth muscle fate at the expense of chondrocytes. Taken together, our results complement previous studies^36,37^, and provide further evidence that intracellular cholesterol may be an important endogenous signal that helps dictate cNCC fate^51^.

There have been no studies in human NCC to demonstrate that they are capable of cholesterol biosynthesis, however, transcriptional profiling of aggressive mouse and human neuroblastoma cells, a NCC-derived malignancy, have demonstrated increased cholesterol biosynthesis driven by the transcription factor sterol regulatory-element binding protein-2 (SREBP-2)^26^. Lipid droplets have also been identified in migratory and post-migratory trunk NCC in E8.5–9.5 mouse embryos^52^, indicative of a potential cholesterol reservoir. In addition, Smith-Lemli-Opitz syndrome, a rare human condition caused by a defect in 7-dehydrocholesterol reductase, is associated with dysmorphic features affecting the head (eg, microcephaly), face (eg, cleft palate), and extremities (eg, poly- or syndactyly) as well as cardiac and intestinal (aganglionosis) defects that may in part reflect impaired NCC function^53^. Lastly, previous studies in zebrafish carrying mutant HMGCS and HMGCR, critical enzymes in the cholesterol biosynthesis pathway, identified malformations in cranial cartilage due to deficient NCC differentiation^36,54^, consistent with our data using cholesterol inhibitors during chondrogenesis in O9-1 cNCC.

Given the phenotypic variability in our arhinia cohort and our interest in potential environmental modifiers acting in utero, we were also interested in the effects of glucose and cholesterol availability on *Smchd1* expression during chondrogenesis. We observed that *Smchd1* mRNA expression was increased at higher glucose levels, and it was lower at the completion of chondrogenesis in the presence of fatostatin (HG and CG) and fluvastatin (CG). Thus, *Smchd1* expression appears to be sensitive to both glucose availability and cellular cholesterol content (during chondrogenesis). If human *SMCHD1* missense mutations do in fact act in a gain-of-function manner^16^, an increase in *Smchd1* expression driven by higher glucose could conceivably exacerbate the phenotype, whereas a decrease in expression during chondrogenesis could create a milder phenotype (e.g., nasal hypoplasia or anosmia). It is also conceivable that a woman could unknowingly be exposed to statins of natural and fungal origin during pregnancy. Although statins have not been definitively linked to birth defects^55,56^, statin in utero exposure could alter the phenotypic effects of an existing *SMCHD1* mutation, contributing to decreased SMCDH1-mediated repressive activity and variability in human phenotypes among *SMCHD1* mutation carriers^15^.

Overall, our study demonstrates a crucial role for a novel, glucose-mediated modulation of cholesterogenesis that acts as “gatekeeper” of cNCC physiology and function, providing metabolic signals that influence cell proliferation, migration, and differentiation. Cholesterol plays an important role in mouse cNCC cell migration and differentiation toward a chondrogenic or myogenic lineage, an important modulation process that is dampened by supraphysiological concentrations of glucose such as those observed in gestational diabetes. We also demonstrate that the expression of the epigenetic repressor, *Smchd1*, is sensitive to glucose (in O9-1 media) and to cholesterol dosage during chondrogenesis, providing additional confirmation of the link between cholesterol, cNCC physiology, and craniofacial development. Further studies, including whether or not upregulation of cholesterol can rescue these cellular phenotypes, are needed to delineate the mechanistic underpinnings of this cholesterol-mediated regulation of cNCC behavior under conditions of varying glucose availability.

## Materials and methods

### Cell culture

O9-1 cells were a gift from K. Shpargel (UNC-Chapel Hill). Cells were expanded on Matrigel-coated wells at 37 °C, 5% CO_2_ in mouse embryonic fibroblasts (MEF)-conditioned basal media supplemented with 25 ng/mL basic fibroblast growth factor (bFGF, R&D Systems) and 1000 U/mL leukemia inhibitory factor (LIF, Millipore) as previously described^3^. For RNAseq, and cholesterol analyses, cells were seeded at 10–15,000 cells/cm^2^ and harvested 48 h after reaching > 80% confluency.

RNA samples for qPCR and RNAseq were extracted from triplicate cultures of O9-1 cells grown in various substrate conditions and purified using the RNeasy Mini Kit (QIAGEN). RNA concentration was measured with the Qubit™ RNA HS Assay Kit and fluorometer (Invitrogen).

### RNA-seq and weighted gene co-expression network analysis (WGCNA)

Libraries were generated using TruSeq RNA Library Prep Kit v2 (Illumina, RS-122–2001) according to manufacturer’s instructions. Purified libraries were quantified on an Agilent Technologies 2100 Bioanalyzer with an Agilent High Sensitivity DNA Kit. Libraries were sequenced on an Illumina NovaSeq 6000 platform to generate 150 base pair single-end reads. The FastQC software^57^ was used to evaluate the quality of sequencing and reads with a phred-quality score < 20 were discarded. The remaining high-quality reads were aligned to the mouse (mm10) reference genome with the STAR aligner^58^. The featuresCounts utility from the Subread package was used to quantify reads aligning to Gencode v.32 mouse genes and differential expression analysis was performed using DeSeq2^59,60^. Genes with log2 fold change > 1 and Bonferroni-adjusted *p* < 0.05 were considered differentially expressed.

Normalized expression values were obtained using the DeSeq2 median of ratios method^60^. Informative genes for Weighted Gene Co-expression Network Analysis (WGCNA) were selected based on high variability and with normalized expression values > 5 in half of the samples. WGCNA was performed using the blockwiseModule utility with with parameter: soft threshold = 22, networkType = ”signed”, TomType = ”signed”, deepSplit = 2, minClusterSize = 30, cutTreeDynamic = 0.25^22^. Modules with a similarity threshold greater than 0.25 were merged. Genes with module membership > 0.6 for the assigned module were selected for pathway analysis with the R gProfileR software package^61^.

The R software (v 4.1.2) WGCNA package (v 1.71; https://bmcbioinformatics.biomedcentral.com/articles/10.1186/1471-2105-9-559?ref=https://githubhelp.com )^22^ was used to panels in Fig. 1. The correlation between the module eigengene value, which is the first principal component of the gene expression matrix for a given module and represents the gene expression pattern for that module, and the glucose substrate is shown in Fig. 1. Pearson correlation values between module eigengene value and substrate availability; high glucose (HG = 25 mM glucose, 1 mM pyruvate), control glucose (CG = 5.55 mM glucose, 1 mM pyruvate), no glucose (NG = 0 mM glucose, 1 mM pyruvate), and no glucose with 2 × pyruvate (NG2P = 0 mM glucose, 2 mM pyruvate) with adjusted p-values (in parentheses) are shown in each bin; correlation of 1 or -1 indicates a strong positive or negative relationship, respectively. For example, the positive correlation for the blue module indicates that genes within that module have increased gene expression as the substrate changes from HG to NG2P experimental conditions. Conversely, genes assigned to the turquoise module have decreased expression as the substrate changes from HG to NG2P conditions.

### Lipid extraction and cholesterol measurement

Lipids were extracted using the lipid extraction kit (Abcam) per manufacturer’s instructions. Briefly, frozen cell pellets were treated with extraction buffer, and they were centrifuged at 10,000 × *g* for 5 min, and the supernatants were transferred to a clean tube and dried at 37 °C overnight. Extracts were resuspended in 50 µL of resuspension buffer. Total cholesterol levels were measured using the Amplex Red Cholesterol Assay kit (Invitrogen) according to manufacturer’s specifications. Sample fluorescence was measured by excitation at 550 nm and emission detection at 590 nm. Cholesterol levels were normalized to protein levels, and results expressed as percentage of cholesterol levels in HG vehicle conditions.

### Apoptosis and migration assays

Real-time, automated Incucyte live-cell Casp3/7 apoptosis assays^62^ were performed on cultures that were ~ 30% confluent. The assays were conducted using an Incucyte live-cell analysis system (Sartorius) via direct treatment with Incucyte Caspase-3/7 dyes, monitoring by time-lapse imaging (every 2 h for 3d), and apoptosis quantification using the Incucyte Cell-by-Cell Analysis Software Module^63^ (Essen Bioscience).cNCC migration was examined using the Incucyte Scratch Wound Assay^64^. Briefly, O9-1 cells were seeded on Matrigel-coated Incucyte Imagelock plates and cultured in appropriate media until the cell monolayer reached 100% confluence. Wounds were created using the Woundmaker tool to create precise, uniform cell-free zones in the cell monolayer. Wells were imaged every hour for 36 h, and cell migration was quantified using the Incucyte Scratch Wound Analysis Software module^64^ (Essen Bioscience).

### Chondrogenesis

O9-1 cells were seeded on Matrigel-coated wells in basal medium. Monolayer cultures were initially treated with osteogenic medium (α-MEM, 10% FBS, 100 U/mL penicillin, 100 μg/mL streptomycin, 0.1 μM dexamethasone, 10 mM β-glycerophosphate, 50 µg/mL ascorbic acid, and 100 ng/mL BMP2 (vendor) for 3 days. After 3 days, cells were switched to chondrogenic medium (α-MEM, 5% fetal bovine serum (FBS), 1% ITS (vendor), 100 U/mL penicillin, 100 μg/mL streptomycin, 10 ng/mL TGF- β3 (vendor), 50 μg/mL ascorbic acid, 10 ng/mL BMP2 (vendor), 0.1 μM dexamethasone, and 1 mM sodium pyruvate) and cultured for 7 days.

Chondrogenic differentiation was assessed by Alcian blue staining^65^. Medium was removed and cells washed twice with DPBS. Cells were then fixed in 4% paraformaldehyde for 10 min at room temperature. Cells were washed and incubated in Alcian blue solution (Millipore) for 30 min; nuclei were stained with Nuclear Fast Red solution (Abcam). Alcian blue staining was measured by spectrophotometric quantification of cells in 4cm^2^/sample wells at 620 nm.

### Smooth muscle differentiation

O9-1 cells were seeded on Matrigel-coated wells in basal medium. Monolayer cultures were maintained in smooth muscle differentiation medium (DMEM, 10% FBS, 100 U/mL penicillin, and 100 μg/mL streptomycin) for 7 days and fixed for downstream experiments.

To detect smooth muscle differentiation, cells were fixed in 4% paraformaldehyde for 10 min at room temperature, followed by permeabilization with 0.4% Triton X-100/DPBS for 10 min. Cells were blocked with 10% BSA/0.1% Tween 20, incubated with smooth muscle actin antibody (Santa Cruz Biotechnology), and fluorophore tagged secondary antibody (Invitrogen); nuclei were stained with Hoechst 33,342.

### Immunofluorescence

O9-1 cells were fixed in with 4% paraformaldehyde in PBS pH 7.4 for 10 min at RT. Cells were permeabilized with PBS 0.4% Triton X-100 for 10 min. Cells were blocked with 10% BSA PBS 0.1% Tween 20. Cells were incubated with smooth muscle actin antibody (Santa Cruz Biotechnology) in 3% BSA PBS 0.1% Tween 20. Cells were incubated with fluorophore tagged secondary antibody (Invitrogen) in 3% BSA PBS 0.1% Tween 20. Nuclei was stained with Hoechst.

### Real-time quantitative PCR

Total RNA was reverse transcribed with iScript cDNA synthesis kit (Bio-Rad). Real-time quantitative PCR was performed using a CFX96 real-time system with a sso advanced universal SYBR green super mix (Bio-Rad). β-actin expression was used to normalize gene of interest in each sample. Real-time quantitative PCRs were set up using the oligonucleotide primers *β-actin* F 5-CGCATCCTCTTCCTCCCTGG-3’, *β-actin* R 5-GTGGTACCACCAGACAGCAC-3’, *Hmgcs* F 5-TGATCCCCTTTGGTGGCTGA-3’; *Hmgcs* R 5’-AGGGCAACGATTCCCACATC-3’, *Hmgcr* F 5-ATCCTGACGATAACGCGGTG-3’; *Hmgcr* R 5’-AAGAGGCCAGCAATACCCAG-3’, *18S* F 5-AAACGGCTACCACATCCAAG-3’; *18S* R 5’-CGCTCCCAAGATCCAACTAC-3’, *Lss* F 5-GGGCTGGTGATTATGGTGGT-3’; *Lss* R 5’-CTCGATGTGCAAGCCCCA-3’, *Mvd* F 5-ATGGCCTCAGAAAAGCCTCAG-3’; *Mvd* R 5’-TGGTCGTTTTTAGCTGGTCCT-3’, *Smchd1* F 5’-GATGGCCTTGACAGCTCAAAC-3, *Smchd1* 5’-CGCCAAGTAAAACACAGATCCTT-3’, *Srebf2* F 5-GACCGCTCTCGAATCCTCTTATGTG-3’; *Srebf2* R 5’-GTTTGTAGGTTGGCAGCAGCA-3’. Fold change was obtained by calculating 2^−ΔΔCt^.

### Statistical analysis

Data were analyzed using Prism 9 (GraphPad Software). Statistical significance was determined by one-way ANOVA with the Dunnett test for multiple comparisons and two-way ANOVA with Tukey test for multiple comparisons. All experiments were performed at least three times. Data are presented as means ± SD, and the level of significance was set at *p* < 0.05.

## Supplementary Information

Supplementary Information 1.

Supplementary Information 2.

## Data Availability

The datasets supporting the conclusions of this article are available in the Sequence Read Archive (SRA) repository (http://www.ncbi.nlm.nih.gov/sra/), accession number: PRJNA883392.

## References

[1] Clay MR, Halloran MC (2010). Control of neural crest cell behavior and migration: Insights from live imaging. Cell. Adhes. Migr..

[2] Krejci A, Tennessen JM (2017). Metabolism in time and space—exploring the frontier of developmental biology. Development.

[3] Bhattacharya D, Khan B, Simoes-Costa M (2021). Neural crest metabolism: At the crossroads of development and disease. Dev. Biol..

[4] Aberg A, Westbom L, Kallen B (2001). Congenital malformations among infants whose mothers had gestational diabetes or preexisting diabetes. Early Hum. Dev..

[5] Schaefer-Graf UM (2000). Patterns of congenital anomalies and relationship to initial maternal fasting glucose levels in pregnancies complicated by type 2 and gestational diabetes. Am. J. Obstet. Gynecol..

[6] Temple R (2002). Association between outcome of pregnancy and glycaemic control in early pregnancy in type 1 diabetes: Population based study. BMJ.

[7] Yang J, Cummings EA, O'Connell C, Jangaard K (2006). Fetal and neonatal outcomes of diabetic pregnancies. Obstet. Gynecol..

[8] Suzuki N, Svensson K, Eriksson UJ (1996). High glucose concentration inhibits migration of rat cranial neural crest cells in vitro. Diabetologia.

[9] Wang XY (2015). High glucose environment inhibits cranial neural crest survival by activating excessive autophagy in the chick embryo. Sci. Rep..

[10] Yang P, Shen WB, Reece EA, Chen X, Yang P (2016). High glucose suppresses embryonic stem cell differentiation into neural lineage cells. Biochem. Biophys. Res. Commun..

[11] Ishii M (2012). A stable cranial neural crest cell line from mouse. Stem. Cells Dev..

[12] Laberthonniere C (2021). AKT signaling modifies the balance between cell proliferation and migration in neural crest cells from patients affected with bosma arhinia and microphthalmia syndrome. Biomedicines.

[13] Inoue K (2023). DUX4 double whammy: The transcription factor that causes a rare muscular dystrophy also kills the precursors of the human nose. Sci. Adv..

[14] Graham JM, Lee J (2006). Bosma arhinia microphthalmia syndrome. Am. J. Med. Genet. A.

[15] Shaw ND (2017). SMCHD1 mutations associated with a rare muscular dystrophy can also cause isolated arhinia and Bosma arhinia microphthalmia syndrome. Nat. Genet..

[16] Gordon CT (2017). De novo mutations in SMCHD1 cause Bosma arhinia microphthalmia syndrome and abrogate nasal development. Nat. Genet..

[17] Houshmand A, Jensen DM, Mathiesen ER, Damm P (2013). Evolution of diagnostic criteria for gestational diabetes mellitus. Acta Obstet. Gynecol. Scand..

[18] Hauguel S, Desmaizieres V, Challier JC (1986). Glucose uptake, utilization, and transfer by the human placenta as functions of maternal glucose concentration. Pediatr. Res..

[19] Nagaraj R (2017). Nuclear localization of mitochondrial TCA cycle enzymes as a critical step in mammalian zygotic genome activation. Cell.

[20] Nioosha Nekooie-Marnany, R. F., Sophie Féréol, Marine D., Roberto M., Roberta F., Jean-Loup D., and Sylvie D. Glucose oxidation and nutrients availability drive neural crest development. *BioRxiv, *10.1101/2022.09.05.506657

[21] Schell JC (2017). Control of intestinal stem cell function and proliferation by mitochondrial pyruvate metabolism. Nat. Cell Biol..

[22] Langfelder P, Horvath S (2008). WGCNA: An R package for weighted correlation network analysis. BMC Bioinf..

[23] Xiao X, Luo Y, Peng D (2022). Updated understanding of the crosstalk between glucose/insulin and cholesterol metabolism. Front. Cardiovasc. Med..

[24] Theisen MJ (2004). 3-hydroxy-3-methylglutaryl-CoA synthase intermediate complex observed in "real-time". Proc. Nat. Acad. Sci. U S A.

[25] Lindgren V, Luskey KL, Russell DW, Francke U (1985). Human genes involved in cholesterol metabolism: Chromosomal mapping of the loci for the low density lipoprotein receptor and 3-hydroxy-3-methylglutaryl-coenzyme a reductase with cDNA probes. Proc. Nat. Acad. Sci. U S A.

[26] Liu M (2016). Transcriptional profiling reveals a common metabolic program in high-risk human neuroblastoma and mouse neuroblastoma sphere-forming cells. Cell Rep..

[27] Hu Z (2021). Synergistic effect of statins and abiraterone acetate on the growth inhibition of neuroblastoma via targeting androgen receptor. Front. Oncol..

[28] Shao W, Machamer CE, Espenshade PJ (2016). Fatostatin blocks ER exit of SCAP but inhibits cell growth in a SCAP-independent manner. J. Lipid Res..

[29] Graham A, Koentges G, Lumsden A (1996). Neural crest apoptosis and the establishment of craniofacial pattern: An honorable death. Mol. Cell Neurosci..

[30] Kubota T (2004). Apoptotic injury in cultured human hepatocytes induced by HMG-CoA reductase inhibitors. Biochem. Pharmacol..

[31] Gao S (2018). Fatostatin suppresses growth and enhances apoptosis by blocking SREBP-regulated metabolic pathways in endometrial carcinoma. Oncol. Rep..

[32] Gholkar AA (2016). Fatostatin inhibits cancer cell proliferation by affecting mitotic microtubule spindle assembly and cell division. J. Biol. Chem..

[33] Cai Y, Zhao F (2021). Fluvastatin suppresses the proliferation, invasion, and migration and promotes the apoptosis of endometrial cancer cells by upregulating Sirtuin 6 (SIRT6). Bioengineered.

[34] Selleck MA, Scherson TY, Bronner-Fraser M (1993). Origins of neural crest cell diversity. Dev. Biol..

[35] Nguyen BH, Ishii M, Maxson RE, Wang J (2018). Culturing and manipulation of o9–1 neural crest cells. J. Vis. Exp..

[36] Quintana AM, Hernandez JA, Gonzalez CG (2017). Functional analysis of the zebrafish ortholog of HMGCS1 reveals independent functions for cholesterol and isoprenoids in craniofacial development. PLoS One.

[37] Castro VL (2020). Activation of WNT signaling restores the facial deficits in a zebrafish with defects in cholesterol metabolism. Genesis.

[38] Pierret C, Spears K, Maruniak JA, Kirk MD (2006). Neural crest as the source of adult stem cells. Stem. Cells Dev..

[39] Freter S, Fleenor SJ, Freter R, Liu KJ, Begbie J (2013). Cranial neural crest cells form corridors prefiguring sensory neuroblast migration. Development.

[40] Zhang D (2014). The neural crest: A versatile organ system. Birth Defects Res. C Embryo. Today.

[41] Miyazawa H, Aulehla A (2018). Revisiting the role of metabolism during development. Development.

[42] Shyh-Chang N, Daley GQ, Cantley LC (2013). Stem cell metabolism in tissue development and aging. Development.

[43] Shyh-Chang N, Ng HH (2017). The metabolic programming of stem cells. Genes Dev..

[44] DeWane G, Salvi AM, DeMali KA (2021). Fueling the cytoskeleton—links between cell metabolism and actin remodeling. J. Cell Sci..

[45] Bhattacharya D, Azambuja AP, Simoes-Costa M (2020). Metabolic reprogramming promotes neural crest migration via Yap/Tead signaling. Dev. Cell.

[46] Blassberg R, Macrae JI, Briscoe J, Jacob J (2016). Reduced cholesterol levels impair smoothened activation in smith-lemli-opitz syndrome. Hum. Mol. Genet..

[47] Ahlgren SC, Bronner-Fraser M (1999). Inhibition of sonic hedgehog signaling in vivo results in craniofacial neural crest cell death. Curr. Biol..

[48] Sheng R (2014). Cholesterol selectively activates canonical Wnt signalling over non-canonical Wnt signalling. Nat. Commun..

[49] Usami Y, Gunawardena AT, Iwamoto M, Enomoto-Iwamoto M (2016). Wnt signaling in cartilage development and diseases: Lessons from animal studies. Lab. Invest..

[50] Mill C, George SJ (2012). Wnt signalling in smooth muscle cells and its role in cardiovascular disorders. Cardiovasc. Res..

[51] Nourse JL (2022). Piezo1 regulates cholesterol biosynthesis to influence neural stem cell fate during brain development. J. Gen. Physiol..

[52] Patel AV, Johansson G, Colbert MC, Dasgupta B, Ratner N (2015). Fatty acid synthase is a metabolic oncogene targetable in malignant peripheral nerve sheath tumors. Neuro. Oncol..

[53] Kelley RI, Hennekam RC (2000). The smith-lemli-opitz syndrome. J. Med. Genet..

[54] Signore IA (2016). Inhibition of the 3-hydroxy-3-methyl-glutaryl-CoA reductase induces orofacial defects in zebrafish. Birth Defects Res. A Clin. Mol. Teratol..

[55] Chang, J. C. *et al.* Perinatal outcomes after statin exposure during pregnancy. *JAMA Netw Open* **4**, e2141321, 10.1001/jamanetworkopen.2021.41321 (2021). 10.1001/jamanetworkopen.2021.41321 PMC8719244 34967881

[56] Vahedian-Azimi A, Makvandi S, Banach M, Reiner Z, Sahebkar A (2021). Fetal toxicity associated with statins: A systematic review and meta-analysis. Atherosclerosis.

[57] Andrews, S. FastQC: A quality control tool for high throughput sequence data. (2010).

[58] Dobin A (2013). STAR: Ultrafast universal RNA-seq aligner. Bioinformatics.

[59] Liao Y, Smyth GK, Shi W (2014). featureCounts: An efficient general purpose program for assigning sequence reads to genomic features. Bioinformatics.

[60] Love MI, Huber W, Anders S (2014). Moderated estimation of fold change and dispersion for RNA-seq data with DESeq2. Genome Biol..

[61] Kolberg, L., Raudvere, U., Kuzmin, I., Vilo, J. & Peterson, H. gprofiler2—an R package for gene list functional enrichment analysis and namespace conversion toolset g: Profiler, *F1000Res,* **9**, 10.12688/f1000research.24956.2(2020). 10.12688/f1000research.24956.1 PMC7859841 33564394

[62] Hanson KM, Finkelstein JN (2019). An accessible and high-throughput strategy of continuously monitoring apoptosis by fluorescent detection of caspase activation. Anal. Biochem..

[63] Granger JE, Appledorn DM (2021). Kinetic measurement of apoptosis and immune cell killing using live-cell imaging and analysis. Methods Mol. Biol..

[64] Kobelt D, Walther W, Stein US (2021). Real-time cell migration monitoring to analyze drug synergism in the scratch assay using the incucyte system. Methods Mol. Biol..

[65] Ishii M (2005). Combined deficiencies of Msx1 and Msx2 cause impaired patterning and survival of the cranial neural crest. Development.

