# Supplementary material for: Cholesterol biosynthesis modulates differentiation in murine cranial neural crest cells
Source: Sci Rep. 2023 May 1;13:7073. doi: 10.1038/s41598-023-32922-9 (PMC10151342; doi:10.1038/s41598-023-32922-9)
Supplement: Supplementary file 1 — Supplementary Information 1. [file 41598_2023_32922_MOESM1_ESM.pdf]

Cholesterol biosynthesis modulates differentiation in murine cranial neural crest cells

Florencia Pascual*[1], Mert Icyuz*[1], Peer Karmaus[2], Ashley Brooks[3], Michael B. Fessler[2], and Natalie D. Shaw[1]

***Co-first authors**

Supplementary Figure S1. Glucose availability impacts cNCC transcriptome. A-B, Clustering analysis in turquoise module showed cell cycle and DNA repair pathways. C-D, Clustering analysis in blue module revealed sphingolipid and glycosphingolipid metabolism.

Supplementary Table S1. Intersections of high confidence WGCNA genes and DE genes within blue and turquoise modules and their relative differential expression in CG vs HG, NG vs HG, NG vs CG, NG2P vs HG, NG2P vs CG, NG2P vs NG conditions.

Supplementary Figure S2. Cholesterol biosynthesis plays a role in cNCC migration. Cell migration of cNCC cultured in HG, CG, NG, and NG2P conditions in the absence (vehicle) or presence of 10 μM fatostatin or fluvastatin was examined in an Incucyte live-cell analyzer via a scratch-wound assay. n = 4 per group; multiple images per well were collected every hour for 36h. Images at 6hrs interval are displayed. Panel A is HG, panel B is CG, panel C is NG, panel D is NG2P.

Supplementary Figure S1

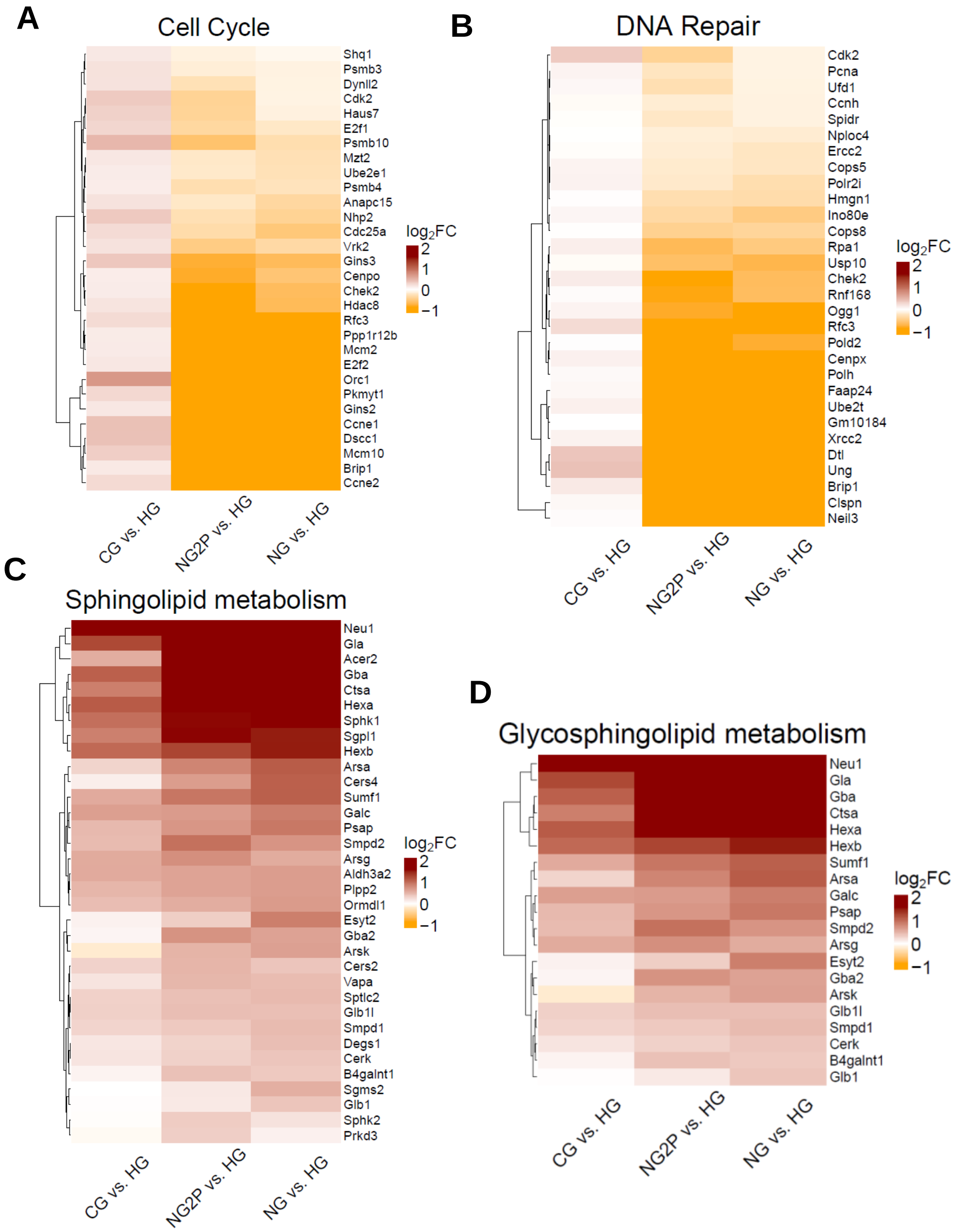

Supplementary Figure S1. Glucose availability impacts cNCC transcriptome. A-B, Clustering analysis in turquoise module showed cell cycle and DNA repair pathways. C-D, Clustering analysis in blue module revealed sphingolipid and glycosphingolipid metabolism.

Supplementary Figure S2

Supplementary Figure S2. Cholesterol biosynthesis plays a role in cNCC migration.
Cell migration of cNCC cultured in HG, CG, NG, and NG2P conditions in the absence (vehicle) or presence of 10 μM fatostatin or fluvastatin was examined in an Incucyte live-cell analyzer via a scratch-wound assay. n = 4 per group; multiple images per well were collected every hour for 36h. Images at 6hrs interval are displayed. Panel A is HG, panel B is CG, panel C is NG, panel D is NG2P.

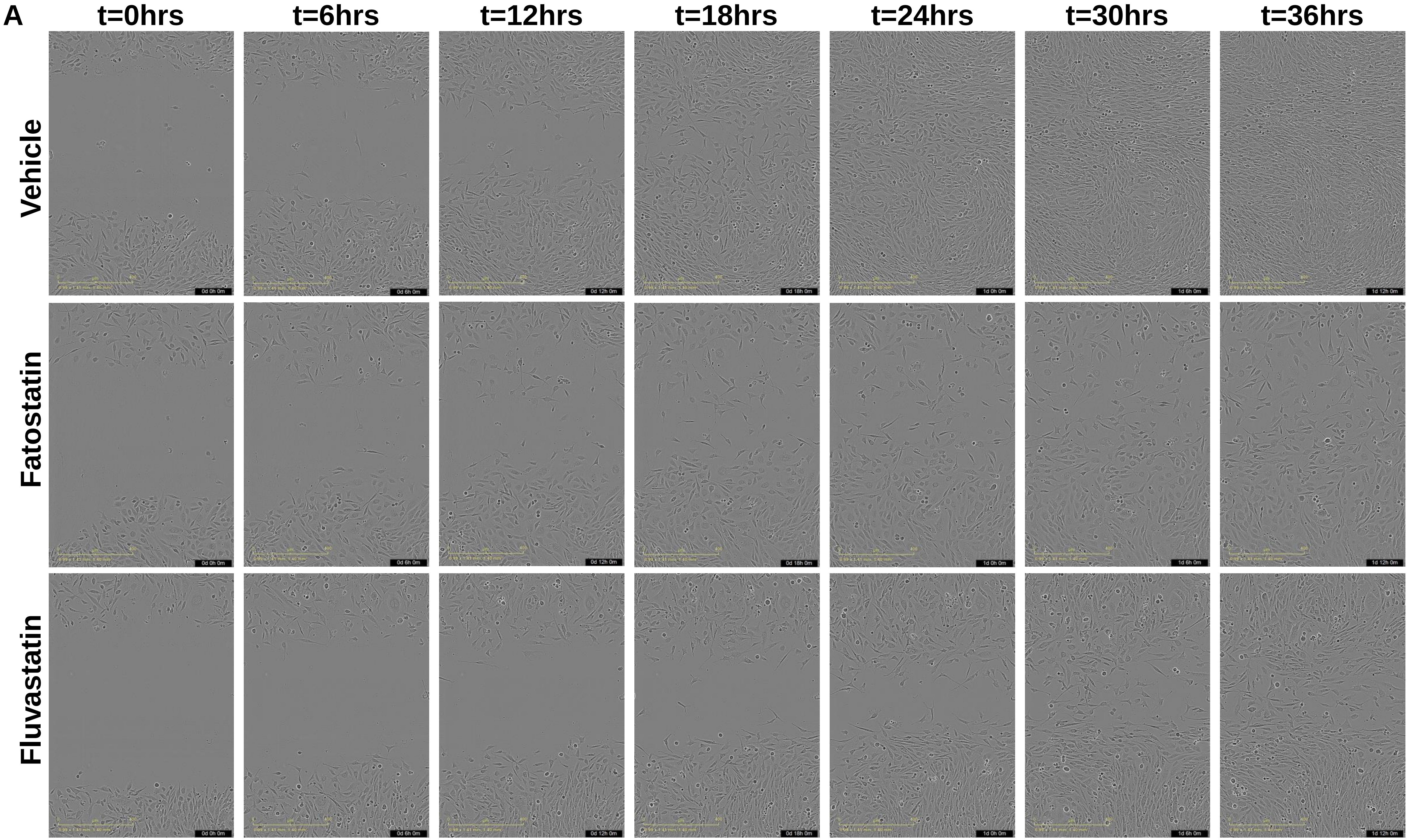
A
t=0hrs
t=6hrs
t=12hrs
t=18hrs
t=24hrs
t=30hrs
t=36hrs
Vehicle
Fatostatin
Fluvastatin

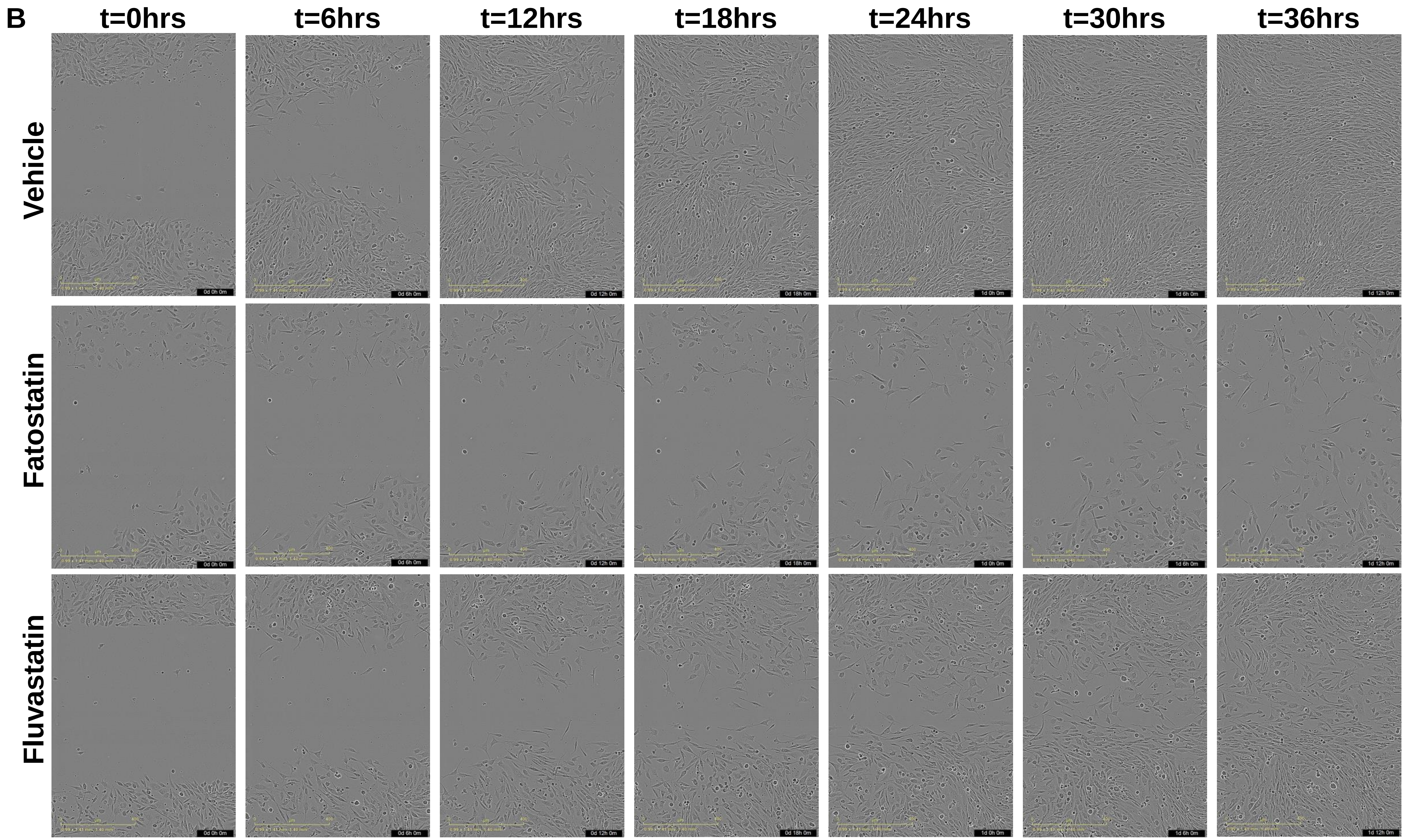
B
t=0hrs
t=6hrs
t=12hrs
t=18hrs
t=24hrs
t=30hrs
t=36hrs
Vehicle
Fatostatin
Fluvastatin

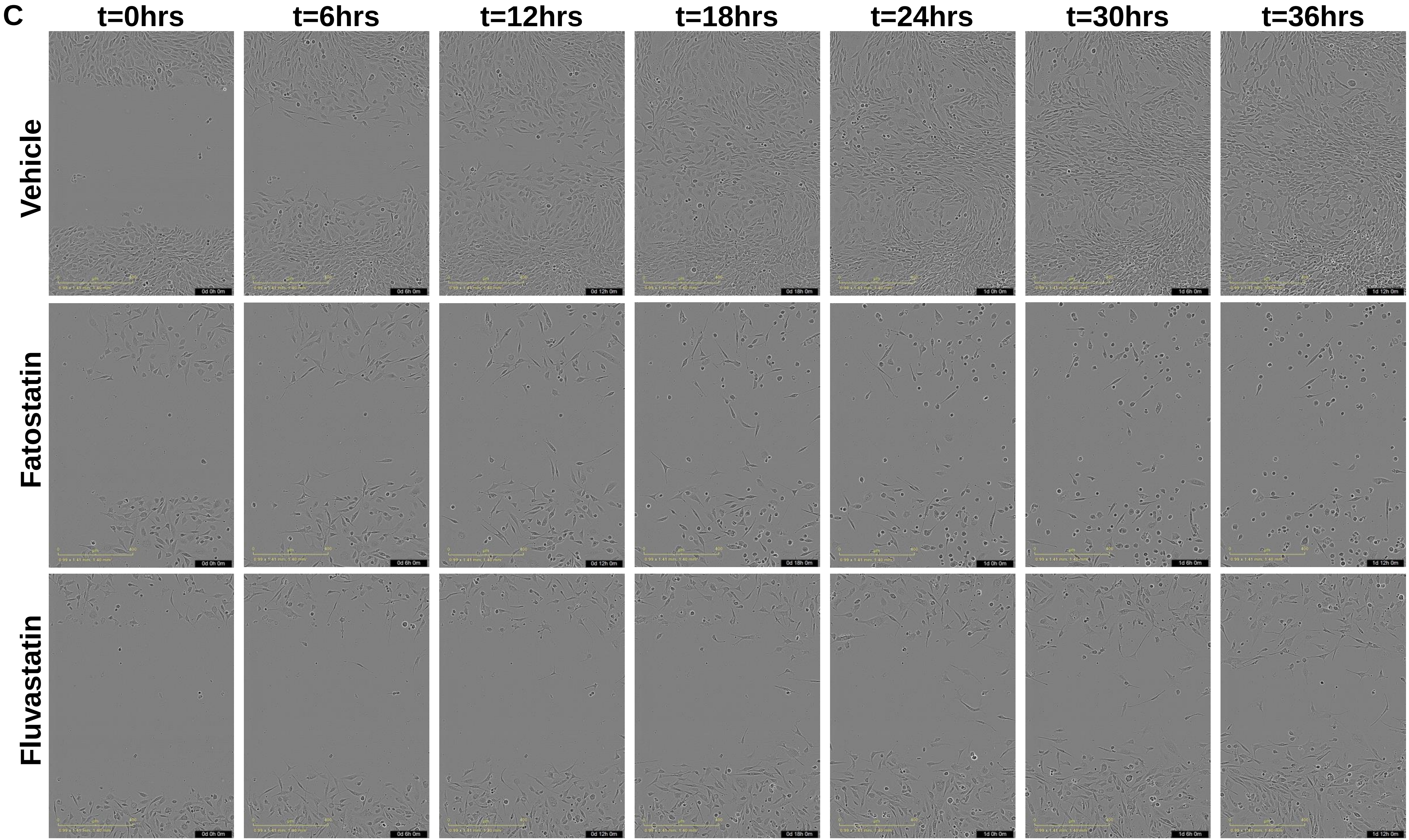
C
t=0hrs
t=6hrs
t=12hrs
t=18hrs
t=24hrs
t=30hrs
t=36hrs
Vehicle
Fatostatin
Fluvastatin

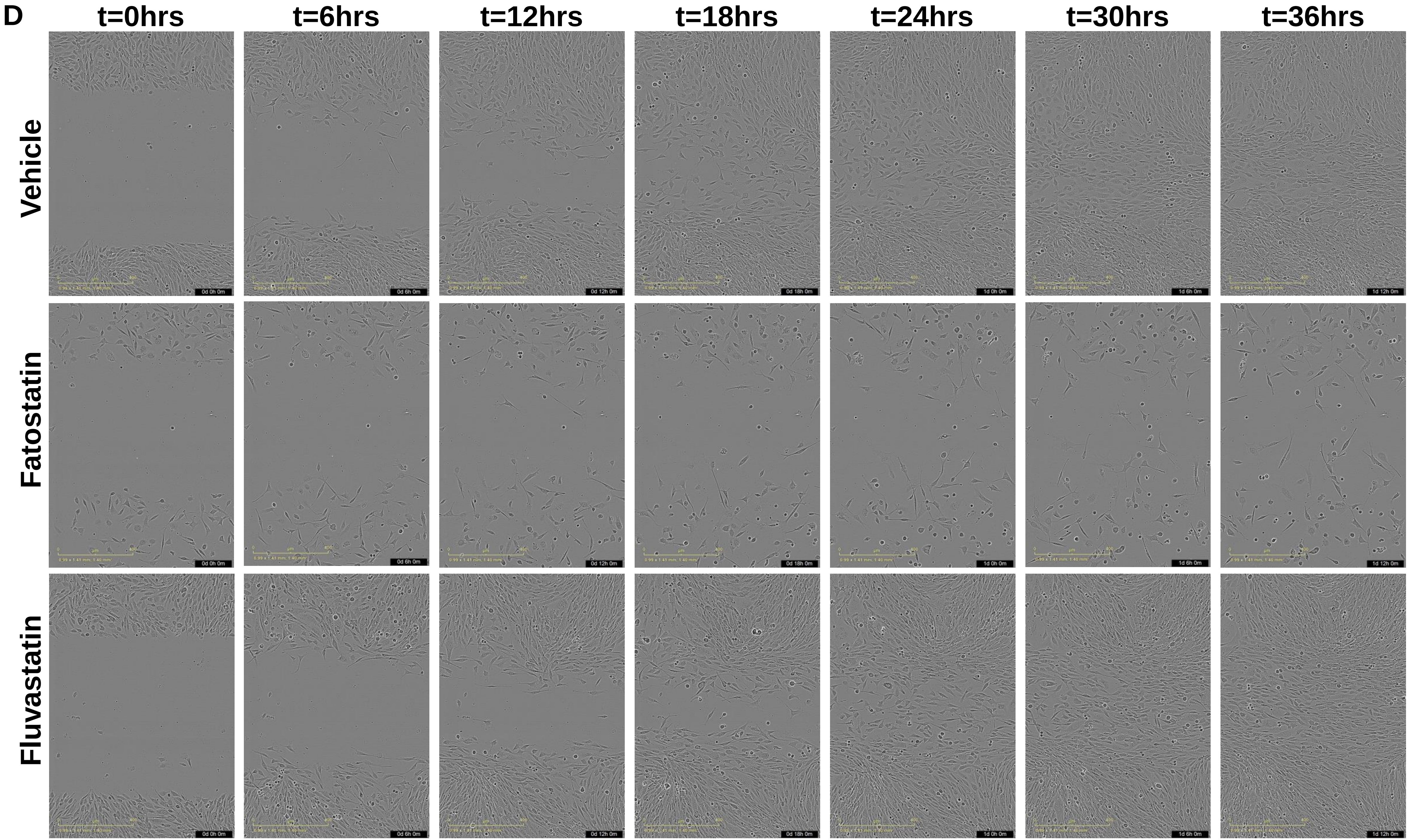
D
t=0hrs
t=6hrs
t=12hrs
t=18hrs
t=24hrs
t=30hrs
t=36hrs
Vehicle
Fatostatin
Fluvastatin
